# Hemolytic uremic syndrome and *Clostridium difficile* colitis

**DOI:** 10.3402/jchimp.v2i3.19064

**Published:** 2012-10-15

**Authors:** Maryam Keshtkar-Jahromi, Mahsa Mohebtash

**Affiliations:** Department of Medicine, Medstar Union Memorial Hospital, Baltimore, MD, USA

**Keywords:** hemolytic uremic syndrome, Clostridium difficile, colitis

## Abstract

Hemolytic uremic syndrome (HUS) can be associated with different infectious etiologies, but the relationship between pseudomembranous colitis and HUS was first described in the 1970s in some childhood patients. There is very limited published literature on *Clostridium difficile*-associated HUS. We report a case of *C. difficile*-related HUS in an adult patient and provide a review of the literature.

Hemolytic uremic syndrome (HUS) can be associated with different infectious etiologies (*Escherichia coli* O157:H7, *Salmonella typhi*, *Shigella dysenteriae*, *Yersinia pseudo tuberculosis*, *Campylobacter jejuni*, *Streptococcus pneumonia*) (1). *E. coli* O157:H7 is the most commonly identified etiology in childhood HUS (<5 years) (2). Infections with Shiga toxin–producing *E. coli*, of which *E. coli* O157 is the most well-known serotype, have been recorded in many regions—including North America, Western Europe, Japan, Central and South America, the Middle and Far East, Africa, and Australia. A possible relationship between pseudomembranous colitis (PMC) and HUS was first described in the late 1970s (3, 4), but *Clostridium difficile* was not known to be the causative agent of PMC at that time. After *C. difficile* was determined to be the causative agent for PMC, some cases of HUS related to PMC in children were reported (5, 6). Only two cases of *C. difficile*-related HUS in adults have been reported (1, 7). We report a case of *C. difficile*-related HUS in an adult patient and provide a review of the literature.

## Case report

The patient was a 62-year-old woman with medical history of chronic anemia who presented with watery diarrhea for 2 weeks associated with nausea, vomiting, and abdominal pain. She had no history of recent hospitalization or antibiotic usage. She had a history of significant weight loss and an all work up for malignancy was reported negative by her primary care physician. In the physical examination, she had a low blood pressure with diffuse abdominal pain. Laboratory studies were significant for a hematocrit of 13, lactate dehydrogenase of 974 U/L, low haptoglobin of < 31 (Nl 31–200), and a negative comb's test. Her creatinine was 13.4 mg/dL. PT/PTT/INR was within the normal limit. Her platelet count was 192,000/L on admission, but dropped to a nadir of 20,000/L after 3 days. A review of peripheral blood smear demonstrated a moderate number of schistocytes. A stool sample was positive for *C. difficile* toxin A by enzyme immunoassay. Stool assay for Shiga-like toxin was negative by PCR, and stool cultures returned negative for *E. coli* O157:H7 and other enteric pathogens. Serum ADAMTS13 activation was 61% (NL > 67%), but ADAMTS13 inhibitor antibody was negative. Of note, ADAMTS13 activity and inhibitor were sent after the initiation of plasmapheresis. Antinuclear antibody was positive, but all other work up for autoimmune disease was reported negative. A diagnosis of *C. difficile* colitis associated with HUS was made; the patient received multiple blood transfusions, plasmapheresis, dialysis, and steroids. Initially, she was started on broad-spectrum antibiotics for possible sepsis, but later her regimen was narrowed down to oral vancomycin. She recovered after 1 month of hospitalization and was discharged with a hematocrit of 32, a platelet count of 140, creatinine of 2.3 (baseline before admission), and baseline mental status.

## Discussion

This is the third reported case of *C. difficile* colitis associated with HUS in an adult (Table 1). All cases were female. All had hemolytic anemia, renal failure, and thrombocytopenia, and all received standard treatment for *C. difficile* colitis. Plasmapheresis and hemodialysis were part of the treatment for two patients. Our patient also received steroids. None of the cases occurred in an outbreak setting. All cases survived without any residual renal or neurologic deficit. *E. coli*-associated HUS has a poorer prognosis in adults compared to children, but prognosis for *C. difficile*-associated HUS might be better in adults than in children.

**Table 1:** Reported cases of *C. difficile*-associated HUS in adults

Case	Age	Sex	Diarrhea type	Confusion	Hemolytic anemia	Acute renal failure	Thrombocytopenia	Treatment	Outcome
Mogyorosi A, 1997	51	Female	Non bloody	Yes	Yes	Yes	Yes	Oral and rectal vancomycin	Recovery
Mbonu CC, 2003	46	Female	Bloody	No	Yes	Yes	Yes	Oral metronidazole, plasmapheresis, hemodialysis	Recovery
Current Case, 2012	62	Female	Non bloody	Yes	Yes	Yes	Yes	Intravenous metronidazole, plasmapheresis, hemodialysis, and steroid	Recovery

The pathogenesis of *C. difficile*-associated HUS is not well defined. It has been speculated that *C. difficile* cytotoxins A and B bind to specific receptors on colonic cell membranes and induce apoptosis leading to mucosal breach, access of cytotoxins to circulation, microvascular damage, and release of proinflammatory cytokines (7). The role of *C. difficile* cytotoxin on renal vasculature and HUS presentation needs further study (7).

## Conclusion

We suggest adding *C. difficile* to the list of possible causes of HUS in adults and children and recommend testing for *C. difficile* as part of an initial work-up for diarrheal HUS patients. The possible role of *C. difficile* in HUS deserves further investigation.
